# The geographic distribution of breast cancer incidence in Massachusetts 1988 to 1997, adjusted for covariates

**DOI:** 10.1186/1476-072X-3-17

**Published:** 2004-08-03

**Authors:** T Joseph Sheehan, Laurie M DeChello, Martin Kulldorff, David I Gregorio, Susan Gershman, Mary Mroszczyk

**Affiliations:** 1 Department of Community Medicine and Health Care, University of Connecticut School of Medicine, 263 Farmington Ave, Farmington, CT, USA 06030-6325; 2 Department of Ambulatory Care and Prevention, Harvard Medical School and Harvard Pilgrim Health Care, 133 Brookline Ave, 6^th ^Floor, Boston, MA, USA 02215; 3 Massachusetts Cancer Registry, Massachusetts Department of Public Health, 2 Boylston St, 6^th ^Floor, Boston, MA, USA 02116

## Abstract

**Background:**

The aims of this study were to determine whether observed geographic variations in breast cancer incidence are random or statistically significant, whether statistically significant excesses are temporary or time-persistent, and whether they can be explained by covariates such as socioeconomic status (SES) or urban/rural status?

**Results:**

A purely spatial analysis found fourteen geographic areas that deviated significantly from randomness: ten with higher incidence rates than expected, four lower than expected. After covariate adjustment, three of the ten high areas remained statistically significant and one new high area emerged. The space-time analysis identified eleven geographic areas as statistically significant, seven high and four low. After covariate adjustment, four of the seven high areas remained statistically significant and a fifth high area also identified in the purely spatial analysis emerged.

**Conclusions:**

These analyses identify geographic areas with invasive breast cancer incidence higher or lower than expected, the times of their excess, and whether or not their status is affected when the model is adjusted for risk factors. These surveillance findings can be a sound starting point for the epidemiologist and has the potential of monitoring time trends for cancer control activities.

## Background

This study is an observational epidemiological investigation of breast cancer incidence in Massachusetts. It examines geographic variations over a ten year period using both purely spatial and space-time models to determine whether observed fluctuations in incidence rates are random or whether fluctuations represent statistically significant deviations from randomness. This study examines whether apparent excesses are stable over time, or are temporary, and also determines whether excesses, high or low, can be accounted for by risk factors such as socioeconomic status (SES) or urban/rural status. This study demonstrates how surveillance data can be analyzed to identify those geographic areas that warrant closer attention, the basis for determining the need for public health action or to aid in assessing the effectiveness of intervention programs [1].

Massachusetts has been included in studies of inter-region and intra-region variability of breast cancer incidence and mortality in the United States. Several of these studies aggregated breast cancer mortality data to the regional or county level [2-8]. Laden and colleagues studied regional variation using 3603 incident cases among nurses from eleven U.S. states [9]. They compared California, the Northeast, and the Midwest to the South; no significant excess incidence of breast cancer was observed in the Northeast. However, because regions and counties are large geographic areas, such studies can miss variability at smaller geographic levels, such as tracts within counties.

A recent study on the geographic distribution of the proportion of late-stage breast cancer cases diagnosed in Massachusetts females between 1982 and 1986 aggregated cases to town, ZIP Code, and census tract levels [10]. The town-, ZIP Code-, and census tract-level analyses all identified approximately the same statistically significantly high area in western Massachusetts. The current study examines the incidence of invasive breast cancer with patients diagnosed between 1988 and 1997.

## Results

### Principal component analysis

The principal components analysis performed on the seven SES variables revealed two components. The two components accounted for about 80% of the variance among the seven economic measures with the loadings of each variable on the two components shown in Table 1. The first component had high positive loadings from median income, median rent, median house value, and percent with at least a high school diploma. This component explained 49.1% of the variance and will be referred to as wealth. The second component had high positive loadings from the percent unemployed, percent working class, and percent below the poverty level. This second component explains an additional 31.0% of the variance and will be referred to as poverty. Although these components are similar, wealth at one end of a spectrum and poverty at the other, they independently contribute to explain SES.

**Table 1:** SES Index. Rotated Component Matrix from the principal component analysis of socioeconomic status (SES) variables.

	Component	
	
SES Variables	Wealth SES	Poverty SES
Percent Unemployed ^b^	0.099	0.932
Median Income ^a^	0.895	-0.294
Median Rent ^a^	0.923	-0.037
Median House Value ^a^	0.835	-0.264
Percent Working Class ^b^	-0.405	0.671
Percent with a High School Diploma ^a^	0.919	-0.081
Percent Below the Poverty Level ^b^	-0.269	0.828

^a^Represented more in the first component. ^b^Represented more in the second component.

Component scores for wealth and poverty were calculated for each tract. The scores were divided into quintiles and used in a Poisson regression to determine their capacity to predict breast cancer incidence. SES variables are scaled so that high wealth scores represent the most wealth and high poverty scores represent the most poverty.

### Poisson regression

The initial Poisson regression of the wealth component showed that category 5, the quintile of the most wealth, had a higher associated risk for breast cancer. There was no increasing or decreasing trend when the fifth category was compared to the other four categories, but they were all lower than the fifth, so categories 1 to 4 were collapsed to create a dichotomous wealth variable. A Poisson regression showed that the highest wealth category had an 8.9% increase in incidence over the combined other categories.

The analysis also revealed that breast cancer incidence was inversely related to poverty level, as shown in Table 2. Those census tracts with the highest poverty levels had an incidence rate 36.9% lower than those in the lowest poverty level; those in the second highest poverty level had an incidence rate 32.2% lower than those in the lowest poverty level, with rates of 29.3% and 19.4% lower for those in categories three and two as compared to those in the lowest poverty level. The analysis also showed that incidence is related to urban/rural status with urban tracts having an incidence rate on average 2.7% higher than rural tracts.

**Table 2:** Wealth and poverty SES. Relative changes in breast cancer incidence associated with 2 levels of wealth and five levels of poverty. For wealth, category 2 represents the highest level of wealth. For poverty, category 5 represents the highest level of poverty. For example, the women in the highest poverty level, 5, had an incidence rate 36.9% lower than those in the lowest poverty level, 1.

	Categories Compared	% Change
Wealth SES	1 – 2	+8.9
Poverty SES	1 – 5	-36.9
	2 – 5	-32.2
	3 – 5	-29.3
	4 – 5	-19.4

### Purely spatial analysis, adjusted for age

**Figure 1:**
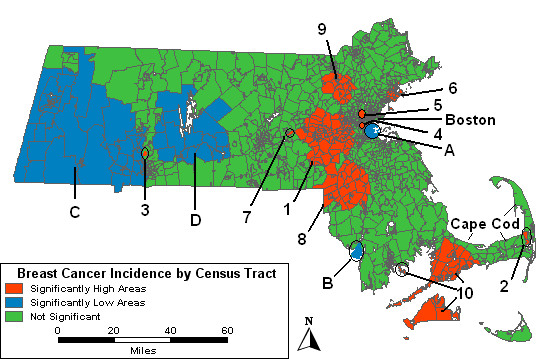
**Purely spatial, age-adjusted. **Purely spatial analysis results for age-adjusted Massachusetts female invasive breast cancer incidence, 1988–1997.

The purely spatial analysis ignores the time of diagnosis. Figure 1 summarizes the purely spatial age-adjusted analysis of breast cancer incidence from 1988 to 1997. Fourteen areas have been identified as excessive in their variation, ten significantly higher and four significantly lower than expected under the null hypothesis. The ten areas of high breast cancer incidence are numbered and the four areas of low incidence are lettered in order of significance, with "1" and "A" having the lowest p-values. For each area of high or low incidence shown in Figure 1, the left hand side of Table 3 contains the relative risks (RR), number of observed cases, and p-value for the purely spatial analysis of the age-adjusted cases. Expected case counts can be calculated from Table 3 by dividing the observed count by the RR. The most statistically significant area of excess, High 1, was found west of Boston. It had 3344 observed cases and a relative risk of 1.15, indicating 15% more cases than would have been expected under the null hypothesis. There are two high areas, labeled "4" and "5", east of High 1. To the west of High 1 is High 7, a single tract with five times more cases than were expected. The second most statistically significant area of excess is High 2 located in the southeast, which includes one tract on Cape Cod, with 2.73 times more cases than expected. High 6 in the northeast shows an elevated risk of 17%. Highs 8 and 9, south and north of High 1, show elevated risks of 15% and 18%, respectively. High 3 in the western part of the state has 2.73 times more cases based on 57 observed and 20.9 expected cases. High 10 in the southeast is elevated by 17%.

**Table 3:** Purely spatial analyses. Age-adjusted female invasive breast cancer statistics for the purely spatial analyses, Massachusetts, 1988–1997. ^a ^Relative Risk. ^b^Area covers more geographic area than in the age-adjusted analysis. ^c^Area covers more geographic area than in the analysis adjusted for SES. ^d ^Area covers less geographic area than in the age-adjusted analysis. ^e ^High 11 is a combination of Highs 2 and 10. *High or low area was not significant for this analysis.

	Age-adjusted only			Adjusted for Urban/Rural Status		
Area	Observed	RR^a^	p-value	Observed	RR^a^	p-value

High						
1	3344	1.15	<0.0001	3344	1.15	<0.0001
2	94	2.73	<0.0001	94	2.40	<0.0001
3	57	2.73	<0.0001	57	2.72	<0.0001
4	126	1.75	0.0002	126	1.74	0.0007
5	276	1.44	0.0003	276	1.43	0.0008
6	1258	1.17	0.0008	1258	1.17	0.0013
7	16	5.05	0.0054	16	5.03	0.0062
8	1354	1.15	0.0060	1354	1.15	0.0097
9	874	1.18	0.0163	874	1.18	0.0190
10	941	1.17	0.0228	997	1.17	0.0120
11^e^	*	*	*	*	*	*
Low						
A	1068	0.74	<0.0001	1068	0.74	<0.0001
B	521	0.77	<0.0001	521	0.76	<0.0001
C	2276	0.89	<0.0001	*	*	*
D	588	0.81	0.0012	*	*	*
E	*	*	*	3600	0.91	0.0004
F	*	*	*	*	*	*

	Adjusted for SES			Adjusted for SES & Urban/Rural Status		

High						
1	*	*	*	*	*	*
2	*	*	*	*	*	*
3	57	3.20	<0.0001	57	3.21	<0.0001
4	*	*	*	*	*	*
5	*	*	*	*	*	*
6	1418^b^	1.16	0.0015	1418^b^	1.15	0.0021
7	16	5.93	0.0013	16	5.93	0.0006
8	*	*	*	*	*	*
9	*	*	*	*	*	*
10	*	*	*	*	*	*
11^e^	1763	1.21	<0.0001	1994^c^	1.21	<0.0001
Low						
A	498^d^	0.73	<0.0001	498^d^	0.73	<0.0001
B	528^b^	0.82	0.0204	528^b^	0.82	0.0226
C	2196	0.91	0.0221	*	*	*
D	588	0.83	0.0243	588	0.83	0.255
E	*	*	*	*	*	*
F	15	0.36	0.0335	*	*	*

Four areas had significantly fewer cases than expected for the ten year period. Low A had 1068 observed cases and a RR of 0.74, indicating about 26% fewer cases than expected. Low B is west of Cape Cod with 521 observed cases and a RR of 0.77. The other two low areas, "C" and "D," in western Massachusetts had 11% and 19% fewer cases than expected, respectively.

### Purely spatial analysis, adjusted for multiple covariates

The remainder of Table 3 shows how the results of the purely spatial analysis of age-adjusted incidence rates within tracts are changed by the inclusion of urban/rural status and SES in the model. When urban/rural status is added to the model, low areas "C" and "D" in rural parts of the state are no longer statistically significant. An area including part of Low D and southward, labeled "E" in Table 3 but not shown in the figures, becomes statistically significant with 9% fewer cases than expected. All other previous findings from the age-adjusted purely spatial analysis are unaffected by the adjustment for urban/rural status.

When the purely spatial model is adjusted for the two SES variables representing wealth and poverty, seven of the high areas are no longer significant. High areas "3," "6" and "7" remain significant. High 6 has expanded to include more tracts and more cases but the RR is about the same. High areas "2" and "10" have merged into a larger area, High 11. Low A covers fewer tracts compared to the age-adjusted analysis, while Low B includes more tracts. A low area, labeled "F" in Table 3 but not shown in the figures, covering Nantucket appears; it includes 15 cases, 64% fewer cases than expected.

**Figure 2:**
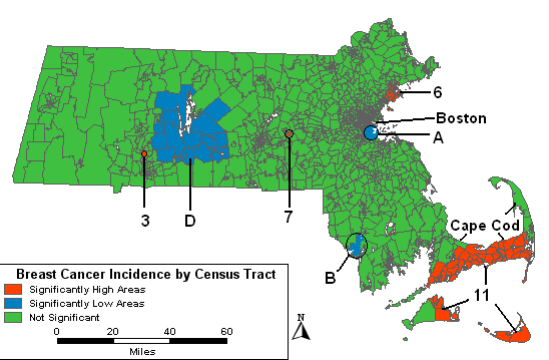
**Purely spatial, multiple adjustments. **Purely spatial analysis adjusted for socioeconomic status and urban/rural status Massachusetts female invasive breast cancer incidence, 1988–1997.

As compared to the analysis adjusting for SES alone, when the two SES variables and urban/rural status are all included in the purely spatial model, the results show little change, except that High 11 is slightly larger in geographic area and low areas "C" and "F" are no longer significant. Figure 2 maps the results of the purely spatial analysis adjusted for urban/rural status and the two SES variables. While three of the original ten high areas remain statistically significant, the total geographic area covered by the high areas has been reduced considerably. One of the four original low areas is no longer statistically significant and despite the slight expansion of Low B, the entire geographic area represented by the low areas has also diminished.

### Space-time analysis

The results of the space-time age-adjusted analysis are somewhat similar to the purely spatial analysis in that seven of the ten areas of excess incidence from the purely spatial analysis were also statistically significantly high in the space-time analysis, along with all four of the low areas. However, as shown in the left side of Table 4, only two of the high areas, "4" and "5," and one of the low areas, Low A, remained statistically significant for the ten-year period. The RRs of these areas were not as elevated in the purely spatial analysis because the space-time analysis captures only the most statistically significantly elevated time periods. However, those areas that were statistically significantly high or low during the whole time period in the space-time analysis had the same RRs in the purely spatial analysis, as they should.

**Table 4:** Space-time analyses. Age-adjusted female invasive breast cancer statistics for space-time analyses of Massachusetts, 1988–1997. ^a^Observed count. ^b^Relative Risk. ^c^Area is shifted east compared to the purely spatial analyses. ^d^Area is significant for 1993–1997 and has dramatically increased geographic area. ^e^Area is significant for 1988–1993 only. ^f^Geographic area increased from the age-adjusted analysis. ^g^Geographic area slightly decreased from the age-adjusted analysis. *High or low area was not significant for this analysis.

		Age-adjusted only			Adjusted for Urban/Rural Status		
	Time Frame	Obs^a^	RR^b^	p-value	Obs^a^	RR^b^	p-value

High							
1	91–97	3639	1.16	<0.0001	3639	1.16	<0.0001
3	89–96	51	3.05	<0.0001	51	3.04	0.0002
4	88–97	126	1.75	0.0078	126	1.74	0.0127
5	88–97	276	1.44	0.0091	276	1.43	0.0154
6	92–95	492	1.35	0.0004	492	1.35	0.0007
7	88–95	*	*	*	*	*	*
8	92–97	841^c^	1.23	0.0034	841^c^	1.23	0.0085
11	93–97	1178	1.28	<0.0001	1178	1.28	<0.0001
Low							
A	88–97	1068	0.74	<0.0001	1068	0.74	<0.0001
B	88–94	1119	0.81	<0.0001	1119	0.81	<0.0001
C	88–96	1951	0.86	<0.0001	1951	0.87	0.0002
D	88–95	450	0.77	0.0069	450	0.78	0.0246
		Adjusted for SES			Adjusted for SES & Urban/Rural Status		
High							
1	91–97	*	*	*	*	*	*
3	89–96	51	3.58	<0.0001	51	3.59	<0.0001
4	88–97	*	*	*	*	*	*
5	88–97	*	*	*	*	*	*
6	92–95	492	1.35	0.0008	492	1.34	0.0005
7	88–95	15	6.95	0.0130	15	6.95	0.0102
8	92–97	2991^d^	1.12	0.0009	2991^d^	1.12	0.0011
11	93–97	1178	1.29	<0.0001	1178	1.31	<0.0001
Low							
A	88–97	600^e^	0.75	<0.0001	600^e^	0.74	<0.0001
B	88–94	1219^f^	0.86	0.0303	1219^f^	0.86	0.0301
C	88–96	1927^g^	0.88	0.0134	*	*	*
D	88–95	*	*	*	*	*	*

**Figure 3:**
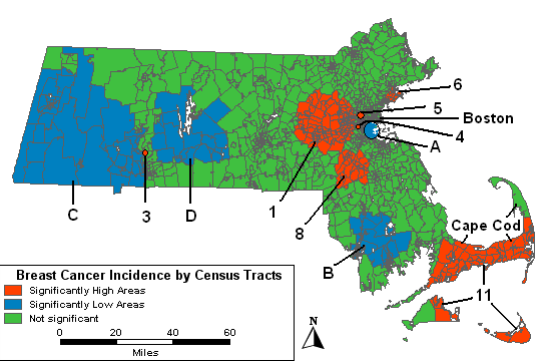
**Space-time analysis, age-adjusted. **Space-time analysis results for age-adjusted Massachusetts female invasive breast cancer incidence, 1988–1997.

Figure 3 displays the results of the space-time age-adjusted analysis. Table 4 shows the time frames, the observed number of cases diagnosed in that time period, the RRs and the p-values. The numbers and letters correspond to approximately the same geographic areas as before. However, while all of the identified areas are still statistically significant at p < .05, they are not necessarily listed in order of statistical significance in Table 4.

The two high areas, "4" and "5", around Boston that were statistically significant in the purely spatial analysis are also statistically significantly high for the entire study period. High 1 was elevated 16% from 1991 to 1997. In northeast Massachusetts, High 6 is statistically significant from 1992 to 1995 with a RR of 1.35. Another high area, High 3, from the purely spatial analysis is also statistically significant in the space-time analysis and represents a single tract in western MA. It is significant from 1989 to 1996 with 51 cases diagnosed and a RR of 3.05. High 9 from the purely spatial analysis was not significant in the age-adjusted space-time analysis. High 11, a large area in the southeast, includes most of High 2 and High 10 from the purely spatial analysis plus some additional tracts that had not been statistically significant before.

Low A in Boston is statistically significant for the entire 10-year period. Low B, in southeastern MA, and Low C, in western MA, remain statistically significantly low from 1988 to 1994, and 1988 to 1996, respectively. Low D, east of Low C, is also statistically significantly low from 1988 to 1995.

### Space-time analysis, adjusted for multiple covariates

Adjusting the space-time analysis for urban/rural status has little effect, except to change the RR and p-values slightly. When the model includes both the wealth and poverty components as covariates, five areas are found to be excessively high, and one of these, High 7, is new to the space-time analyses. This small area had been identified in the purely spatial analysis and remained statistically significant after adjustment for SES. Low D is no longer statistically significant.

**Figure 4:**
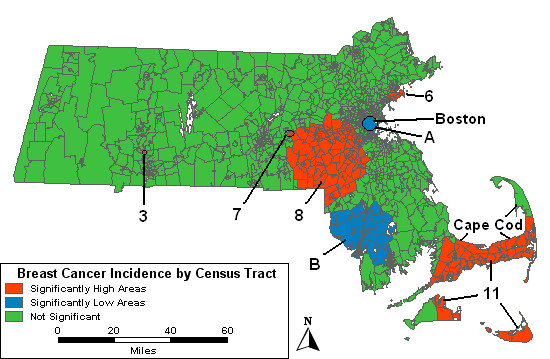
**Space-time analysis, multiple adjustments. **Space-time analysis adjusted for socioeconomic status and urban/rural status Massachusetts female invasive breast cancer incidence 1988–1997.

When adjusting for SES and urban/rural status together, all the high and low areas from the SES and age-analysis, except for Low C, remain significant. These results are displayed in Figure 4.

## Discussion

We examined the possibility of a geocoding bias. Of 46,333 total cases, 4440 were randomly assigned to a census tract located within the reported town. These cases could not be assigned directly to a census tract because their records did not have a residential address. Cancer registries like the MCR collect a patient's usual residence at the time of diagnosis. For patients having only postal box addresses, if the MCR staff were unable to obtain a residential address at the time of diagnosis, we assigned these cases to tracts within the town of the mailing addresses, as described in Methods below. This should not be problematic in larger areas of excess or deficit since error would be confined to a town. In the smaller areas identified by the analyses, all had higher than expected case numbers. We examined the smaller areas of excess to determine how many of the observed cases had been randomly placed into a tract. To estimate how the RRs would be affected, the RRs were re-computed from the purely spatial age-adjusted analysis, while excluding these cases. High areas "2" and "3" did not contain any cases assigned to the tract randomly. In High 4, eight of the 126 cases observed were assigned at random; without these cases, the RR would be 1.63 rather than 1.75. High 5 had 31 of the 276 observed cases assigned randomly; without these cases, the RR would be 1.28 rather than 1.44. Thus, even if none of the randomly assigned cases were assigned to these smaller areas, the relative risks would not be altered substantially. However, MCR has studied High 7 and determined that several large apartment complexes were geocoded as being part of the census tract which makes up High 7 instead of placing it in the correct neighboring tract. Since High 7 does not have many females as part of the population, these geocoding errors may have made a large difference and therefore, this tract is not likely to have high breast cancer incidence.

This study adjusted for age, SES, and urban/rural status. Other known risk factors could possibly explain the high areas uncovered. The following attributable risk percentages for such factors have been reported in the literature: 10.7% due to high alcohol intake, 15.0% due to low beta-carotene intake, 8.6% due to low vitamin E intake, 11.6% due to low levels of physical activity [11], 5.0% due to BRCA1 and BRCA2 mutations [12], 29.5% due to late age at first birth and nulliparity, 9.1% due to family history of breast cancer [13], and 2.5% due to smoking [14]. Patient smoking history data were available but were considered unreliable. These other risk factors were not included in this study since the information was not available in the cancer registry. Also, data on town urban/rural status and the SES variables from the 2000 Decennial Census were not available at the time of the analyses, so we were not able to determine if there were any significant changes over time or how these changes might alter the results. For Massachusetts, we would not expect any significant changes.

This study used aggregated data to compensate for the lack of individual level data and is therefore exposed to the ecological fallacy. Krieger has shown that this is not a serious problem [15]. Also, it would be impossible to perform this study of breast cancer incidence at the point level since no denominator data exist for population counts at the individual address level.

## Conclusions

The current study is part of the surveillance process, an observational epidemiologic investigation to provide reliable statistical modeling of the raw surveillance data so that program evaluation or planning can be focused on those variations in incidence rates with a low likelihood of being random. In the current study it is especially interesting to observe whether geographical areas, high or low, are affected by SES adjustment or not. It is also interesting to observe which geographical areas seem to be high or low for the entire study period, and which are high or low on a temporary basis.

### Recommendations

The first step when studying these high and low areas is to look closely at the geocoded data in these areas to ensure that there were not any geocoding-related errors. An example of a geocoding-related error was High 7. However, this problem should only be an issue in areas that only contain a small number of tracts. Roche et al. have made other suggestions as to what can be done when these areas are found to be truly elevated [16].

Several areas (Highs "1," "4," "5," and "9" and Low C) are no longer significant with the adjustment for SES and urban/rural status. What is it about the high SES in these areas that affects the RR of these areas? It could be that late age at first birth has a substantial contribution since women of high SES delay childbearing to establish their careers. Note that the p-values and RRs are not correlated and both need to be studied. For ease of comparing statistics, refer to Tables 3 and 4.

High areas "3" and "6," as well as Low areas "A" and "B" do not change a great deal with SES adjustment, so these covariates are not having the same effects as they had above. Perhaps a case-control study could be designed on these areas to determine what other risk factors are driving this elevated incidence rate, such as genetic or behavioral factors.

High 6 remained unaffected by adjustment for SES and urban/rural status. However, since it was only temporarily elevated for 4 years, perhaps this area should also be monitored to see if its incidence rate significantly elevates. However, High 11 should be investigated for other factors that may be elevating the risk since it remained elevated for 5 years near the end of the study period.

The low areas should be assessed since cases might be going undetected if sufficient screening programs are not in place. If screening programs are in place, they may need to be altered and improved.

Finally, it is possible for the populations in two different areas to experience the same amounts of cancer; yet if some cancers remain undetected in one area and are diagnosed in the second area, the second area will appear to have a higher incidence. This is why when breast cancer screening is aggressively promoted in an area with worrisome incidence, the cancer rate goes up for a period of time. Furthermore, not all areas have equal access to the latest in diagnostic equipment capable of detecting the most minute cancers or even pre-cancerous cells. A patient's cancer might be diagnosed if she went to a facility with the latest diagnostic equipment, but might not be diagnosed if she had gone to a different place. Incidence may also reflect not just differences in diagnosis rates, but also differences in treatment patterns. If women or physicians in one area tend to opt for more lumpectomies without radiation, the patients still have a chance to develop later cancers in that breast; if women or physicians in another area tend to opt for lumpectomies with radiation or mastectomies for the same stage of disease, they will not be having any subsequent cancers in that breast [17].

## Methods

### Data

Cases are from the Massachusetts Cancer Registry (MCR), and include all invasive breast cancer cases diagnosed in female residents between 1988 and 1997 (n = 46,333), using the standard definition of invasive breast cancer[18]. Annual reports on MCR data completeness, methods and other issues can be found at the cited website [19]. Each case record included the town, ZIP Code, and census tract of the patient's usual residence at the time of diagnosis, as well as the age at diagnosis, date of diagnosis, race, and stage of disease at diagnosis. Of the 46,333 patients, 43,529 were white females, 1071 black, 6 American Indian, Aleutian, or Eskimo, 115 Chinese, 17 Japanese, 129 other Asian and Pacific Islander, 119 other non-white, and 1347 of unknown race. Race was not included as a covariate in this study due to the large number of unknown. Age was included as a covariate and was divided into fourteen 5-year categories starting with age 15 years up through age 84, with all ages 85 years and above as the fifteenth age group.

### Aggregation unit

Census tracts were used to aggregate cases because they are more uniform than towns or Zip Codes in population size, provide a more sensitive analysis of densely populated areas, and are more homogeneous in their resident characteristics. However, 12.6% (n = 5832) of the cases diagnosed in 1988–1997 could not be assigned a reliable residential census tract because of inaccuracies or omissions in the address information provided to the MCR. In most of these cases, a mailing address had been provided and, even after extensive research, MCR staff could not assign a reliable residential address for the patient at the time of diagnosis. To assign the unassigned cases to census tracts, we compared town and census tract boundaries for 1196 four-digit census tracts. About 90 of the census tract codes included 2-digit suffixes to designate 2, 3, 4, or 5 distinct tracts. In this study, only the first four digits of such tract codes were used, thus combining some separate tracts into one. For example, census tracts 6002.01 and 6002.02 were combined into the 4-digit tract 6002. 1990 Census block numbering areas in Massachusetts are treated here as if they were census tracts. Nearly all of the 1196 four-digit 1990 tracts could be related to town boundaries, with one or more tracts located entirely within a town, or one or more towns entirely within a census tract. Two census tracts overlapped parts of multiple towns and for these it was possible to estimate the proportion of the total tract population living in each town. Unassigned cases were then assigned as follows: for towns completely contained in one tract, cases were assigned to that tract, accounting for 1392 cases. For a town containing two or more census tracts, cases were randomly assigned to tracts proportionate to the town's female population, accounting for 4440 cases. The allocation of cases should therefore be free from systematic error and any error should be localized to a particular town, while the broader state patterns remain correct.

### Statistical analyses

The spatial scan statistic [20] was used to perform purely spatial and space-time analyses. Under the null hypothesis, the incidence of breast cancer follows a Poisson distribution and the probability of a case being diagnosed in a particular location is proportional to the covariate-adjusted population in that location. Tract population data are from the 1990[21] and 2000 Decennial Censuses [22]. This population data is an excellent match to the MCR data since both the Census and the MCR ask patients for their "usual residence." Therefore the numerator and denominator are created in the same way.

Properties that make the spatial scan statistic suitable for geographic surveillance are: 1) it takes into account the uneven geographic distribution of cases and population densities, 2) it does not make assumptions about cluster size or location, 3) it adjusts for multiple testing – a common problem in testing multiple combinations of cluster locations and sizes, 4) it identifies the spatial or space-time locations where the null hypothesis is rejected, and 5) it can detect multiple clusters [10].

Purely spatial analyses were performed first, ignoring time. The maximum spatial cluster size was first set to include up to 50% of the population for both excesses and deficits and then set at 10%, to test for excesses and deficits separately because testing at the 10% level can identify smaller, more defined areas. However, each area identified in an analysis had a likelihood associated with it that was compared to the 9,999 likelihoods from the initial 50% maximum spatial size test, thus accounting the inference for a multiplicity of statistical tests. Space-time analyses were then performed to determine whether the clusters from the purely spatial analysis were long term or temporary. The maximum temporal cluster size was set at 90% and also included purely spatial clusters with a temporal size of 100% for all space-time analyses. This allows for the entire time period all the way down to the smallest amount of time to be considered as the time window, ten years down to one year.

All SaTScan analyses were performed on age-adjusted population counts. To calculate the age-adjusted expected counts, the 1990 and 2000 female population counts were combined into a weighted average of the two based on the years being analyzed, 1988 through 1997. This was done for each age group within each tract. The natural log of this weighted average was entered as the offset variable in a Poisson regression in SAS [23]. There were a few tracts with a zero population for a certain age group; for these, a population of one was entered so a log could be taken. The Poisson regression included age as a class variable and the number of cases within each tract and age group as the dependent variable to calculate the age-adjusted expected counts. The expected counts were aggregated across age for all SaTScan analyses. This method was verified by entering age as a covariate in a SaTScan analysis with the same results. Although SaTScan accurately estimates age-adjusted incidence rates, when other covariates enter the model, SaTScan computes the interaction of each of the 15 age categories with each covariate. In the current study, that would mean estimating 14 × 1 × 1 × 4 = 56 interaction terms because three risk factors were used as covariates. By first computing the age-adjusted counts in SAS, the number of interactions estimated by SaTScan is reduced to 8.

After identifying statistically significant geographic areas and determining whether they were long term or temporary, the next step was to determine whether these areas would change when the model was adjusted for known risk factors. Socioeconomic factors have long been identified as risk factors for breast cancer [9,24,25]. Urban/rural status has also been used as a covariate when studying breast cancer [4,26-29].

An SES index was created following the method of Yost et al [30]. Yost and colleagues included the following variables from the U.S. Decennial Census in a principal component analysis (PCA): percent unemployed, percent working class, percent below the Federal poverty level, median income, median rent, median house value, and percent with at least a high school diploma. We used the same variables from the 1990 Census [21] in a PCA, described in the results section. A PCA factors the matrix of correlations between all pairs of variables into seven uncorrelated components with the hope that a smaller number of these components will account for 70 to 80% of the variation contained in the matrix of correlations among the original seven variables. Instead of using seven highly correlated SES variables as covariates, PCA can reduce the number of covariates to one or two without loss of information. A PCA was also performed on a similar group of variable from the 1990 Census based on what Krieger [24] used to create a SES index, however it did not account for as much of the variance as the variables used by Yost [30].

A variable indicating the urban or rural status of each tract was derived from data available on the Massachusetts Institute for Social and Economic Research (MISER) [31]. An urban area consists of one or more contiguous census blocks with a population density of at least 1,000 persons per square mile, while all other areas are designated as rural [32]. We assigned all tracts entirely within a town the same classification as that town, regardless of their individual populations. Tracts that included several towns were classified as rural since all towns within such tracts were also classified as rural. The capacity of SES and urban/rural status to predict incident cases within census tracts was tested in Poisson regression models, using the GENMOD procedure in SAS [23].

SaTScan [33] created files listing census tracts and the number of the high and low areas they belonged to. These files were brought into Maptitude [34] to create the figures.

## Authors' contributions

TJS: PI, responsible for design, funding, of project with overall responsibility for implementing the project, including the final paper. LMD: Principal data analysis, responsible for final checks on accuracy of data and all analyses, including their written interpretation. MK: Co-PI, assisted in the overall design of the project and close collaborator on all analyses. MK also assisted a great deal in editorial decisions of drafts and of the final paper. DG: Co-PI, assisted in the design of the project, including funding and implementation. DG also consulted on editorial and technical issues. SG: Director of MCR, she was responsible for the overall integrity of the data, and ensuring that use of the data corresponded to the policies of the Massachusetts Dept of Public Health. She also assisted with editorial help on all drafts. MM is responsible for the geocoding of the data in the MCR. She was a constant help to this project as we tried multiple approaches to our analyses. MM was also very helpful in clarifying limitations of the work and also provided many editorial suggestions. All authors read and approved the final manuscript.
